# Tissue Stem Cell-Based Therapies in Parkinson’s Disease: A Scoping Review of Therapeutic Mechanisms and Translational Outcomes

**DOI:** 10.3390/cells14110822

**Published:** 2025-06-01

**Authors:** Emily Cueva, Andrea Wiesheu, Zaira Sordo, Jailene González, Sabine Falconi, Jose A. Rodas, Jose E. Leon-Rojas

**Affiliations:** 1 NeurALL Research Group, Quito 170157, Ecuador; emy.jul@hotmail.com (E.C.); anwiesheuar@uide.edu.ec (A.W.); solzai033@gmail.com (Z.S.); jailene2019@hotmail.com (J.G.); sabine.falconig@ug.edu.ec (S.F.); 2 School of Psychology, University College Dublin, D04 V1W8 Dublin, Ireland; josea.rodasp@gmail.com; 3 Escuela de Psicología, Universidad Espíritu Santo, Samborondón 092301, Ecuador; 4 Cerebro, Emoción y Conducta (CEC) Research Group, Medical School, Universidad de las Américas (UDLA), Quito 170124, Ecuador

**Keywords:** Parkinson’s disease, tissue stem cell therapy, neuroregeneration, dopaminergic neurons, neuroinflammation

## Abstract

(1) Background: Parkinson’s disease (PD) is a progressive neurodegenerative disorder characterised by dopaminergic neuronal loss. Tissue stem cell-based therapies have emerged as promising candidates for disease modification and symptomatic relief. This scoping review aims to systematically synthesise the literature on tissue stem cell therapies for PD across cellular, animal, and human studies, with a focus on transplantation strategies, mechanisms of action, and therapeutic outcomes. (2) Methods: We identified 1017 records by querying PubMed, Scopus, Cochrane, and the Virtual Health Library. After screening and applying eligibility criteria, 33 experimental studies were included. Data were extracted on study design, tissue stem cell source, type of subject, and therapeutic effects. (3) Results: Most studies (n = 25) involved animal models, with a minority (n = 8) focusing on human applications. Tissue stem cell therapies showed potential to promote dopaminergic differentiation, reduce inflammation and apoptosis, and improve behavioural and motor outcomes. Autologous transplants yielded a higher safety and efficacy compared to allogeneic ones. The beneficial mechanisms of tissue stem cells included neurotrophic support, mitochondrial protection, modulation of the gut–brain axis, and α-synuclein clearance. (4) Conclusions: Tissue stem cell therapies represent a promising approach for PD. However, standardised protocols and long-term safety assessments are essential to optimise their translational potential.

## 1. Introduction

Parkinson’s disease (PD) is a progressive neurodegenerative disorder marked by the loss of dopaminergic neurons, leading to debilitating motor and cognitive symptoms [1]. Standard pharmacological treatments, including levodopa and dopaminergic agonists, primarily provide symptomatic relief without altering the course of the disease [2]. This limitation has fuelled interest in innovative therapies, particularly tissue stem cell therapy, which has shown promise in experimental models for not only symptom management but potentially modifying the progression of the disease [3]. Tissue stem cell therapy may have neuroprotective effects and the potential to replace lost neurons, restore damaged neural circuits, secrete neurotrophic factors, modulate inflammatory responses, and offer long-term therapeutic benefits for PD patients [4].

Our manuscript presents a scoping review, conducted in accordance with PRISMA-ScR guidelines, with the aim of mapping the current research landscape on tissue stem cell therapies in PD. Our objective is not to evaluate clinical efficacy but rather to synthesise emerging data on cellular sources, transplantation strategies, proposed mechanisms of action, and translational potential across preclinical and clinical studies. By identifying prevailing trends and knowledge gaps, we seek to inform future research directions and facilitate responsible scientific progress in this evolving field.

Tissue stem cells’ potential to restore motor and cognitive functions and secrete neurotrophic factors adds to their therapeutic value, as observed in numerous animal, human, and cellular studies showing improvements in mobility and behavioural functions [3,4]. However, achieving standardised protocols for isolating, differentiating, and delivering tissue stem cells remains a challenge. Nonetheless, animal models have shown important functional improvements, such as reduced rigidity and tremors and enhanced motor recovery following tissue stem cell transplantation [5]. Moreover, some studies report lasting improvements in PD symptoms, with transplanted mesenchymal stem cells (MSCs) and human adipose-derived stem cells (hASCs) contributing to motor function gains, enhanced cognitive abilities, and even increased learning and memory capacities in rodent models [6] and the creation of new dopaminergic neurons in cellular (in vitro) models [7,8,9].

## 2. Materials and Methods

### 2.1. Protocol and Registration

Our review was conducted in accordance with the PRISMA-ScR (Preferred Reporting Items for Systematic reviews and Meta-Analyses extension for Scoping Reviews) guideline, and the study protocol was prospectively registered in the PROSPERO database (CRD4202346441).

### 2.2. Eligibility Criteria

Our inclusion criteria considered all articles from inception until November, 2022; we included articles written in either English or Spanish; only primary study designs were considered (i.e., case–control, cohort, case studies, cross-sectional, randomised clinical trials, experimental in vivo or in vitro studies, case reports, and case series) conducted in cells, animals, or humans that evaluated the therapeutic potential of tissue stem cells for the amelioration of PD’s symptomatology or progression; we decided to include in vitro studies that show the potential new effect of tissue stem cells (i.e., the effect on PD mechanisms or pathophysiology). We excluded secondary design studies (i.e., literature or scoping reviews, systematic reviews, meta-analyses, editorials, and letters to the editor); we also excluded articles that were not subjected to proper peer review and those with models that included types of diseases other than PD; cell cultures that do not simulate neural growth after induced tissue stem cell therapy were also excluded.

### 2.3. Information Sources and Search Strategy

The studies were collected from PubMed, Scopus, Cochrane, and the Virtual Health Library (VHL) databases from inception until 21 November 2022. We used a combination of the following main key terms: Parkinson disease, tissue stem cell, progenitor cell, mother cells, and disease progression. The complete search terms used in each database can be found in the Supplementary Materials.

### 2.4. Selection of Sources of Evidence

After retrieving the articles, a team of six reviewers conducted the systematic review process using Rayyan (https://www.rayyan.ai; accessed on 1 December 2024), a web-based software for systematic reviews used to lower the risk selection and deduplication errors. Our selection process involved two filters conducted by a pair of reviewers blinded to each other’s decisions. The first filter, conducted after deduplication, included the screening of titles, abstracts, and keywords against the aforementioned eligibility criteria; any discrepancies were resolved by a third reviewer and mutual consensus. The second filter was conducted on a similar fashion but involved a complete full-text review, analysing key aspects related to the research question and eligibility criteria. Afterwards, all reviewers convened to analyse the obtained results collectively, engaging in in-depth discussions to ensure a comprehensive understanding of the findings and solve any discrepancies. The reviewers critically evaluated the selected studies and synthesised the data to address the research question. This rigorous process aimed to ensure a thorough evaluation of the potential use of induced tissue stem cell therapy to treat Parkinson’s disease. The involvement of multiple reviewers and the use of blinded screening helped minimise biases, while the detailed assessment and data analysis contributed to producing a robust and comprehensive review.

### 2.5. Data Charting Process and Data Items

All the articles that successfully completed both screening filters were subjected to data extraction on an Excel spreadsheet. We extracted data related to article characteristics (e.g., authors, years, type of study), specific tissue stem cell characteristics (e.g., autologous tissue stem cells, allogeneic tissue stem cells), differentiation and modulation factors (e.g., dopaminergic differentiation, α-synuclein modulation, neuronal differentiation), expression and activity metrics (e.g., MMP-2 expression in MSCs, electrophysiological activity, 3D distribution and electrophysiological activity, Clathrin-mediated endocytosis (CME)), protective and therapeutic effects (e.g., MSCs’ protective effects, MSC-derived factor, anti-apoptotic effects, inhibition of endocytosis, behavioural improvements, improvements in mobility, SPECT scan), treatment and timing aspects (e.g., mitomycin C (MMC) treatment, inflammation and timing, paracrine influence, graft-induced dyskinesia), contextual factors (e.g., relation between gut microbiota and Parkinson’s disease, tyrosine hydroxylase (antibody/immunoreactive), neurotrophic factors, non-tumour formation), and therapeutic outcomes (e.g., long- and short-term therapeutic effects).

### 2.6. Synthesis of Results

To analyse the information provided by the articles, we grouped the studies based on the types of models used (i.e., cells, animals, or humans) and summarised the information according to the previously mentioned topics, including measures used and broad findings. This approach entailed summarising and qualitatively analysing the findings of the selected studies, providing a comprehensive overview of the evidence and enabling a qualitative interpretation of the results.

## 3. Results

### 3.1. Selection of Sources of Evidence

**Figure 1:**
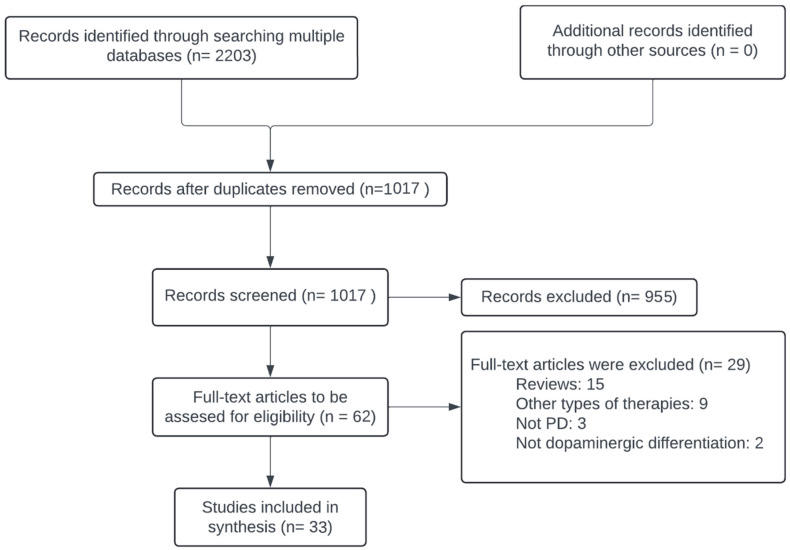
Flowchart of the complete selection process.

After duplicates were removed, a total of 1017 citations were identified from searches of electronic databases. After the removal of duplicates and executing both filters, 33 studies were considered eligible for our scoping review [8,10,11,12,13,14,15,16,17,18,19,20,21,22,23,24,25,26,27,28,29,30,31,32,33,34,35,36,37,38,39,40,41]. The complete selection process is shown in Figure 1.

### 3.2. Characteristics and Results of Individual Sources of Evidence

We classified the selected studies into two distinct groups according to the population studied (animals or humans), and for each classification we evaluated what type of therapy was used (autologous or allogeneic), as well as how these therapies were performed and what similarities were found between studies in the process of differentiation of cells, transplantation, and evaluation of the effects of therapy both in vitro and in vivo; we also extracted the source of the tissue stem cells used (Table 1). Furthermore, we have provided relevant outcome information from each source with the type of subject used in the Supplementary Materials.

### 3.3. Synthesis of Results

A total of 33 studies investigated the connections between the application of tissue stem cells as a curative resource in Parkinson’s disease [8,10,11,12,13,14,15,16,17,18,19,20,21,22,23,24,25,26,27,28,29,30,31,32,33,34,35,36,37,38,39,40,41]. Most studies originated from the United States (n = 6) and China (n = 5), followed by Iran (n = 3), South Korea (n = 3), Italy (n = 2), Mexico (n = 2), Brazil (n = 2), Japan (n = 2), Taiwan (n = 2), and India (n = 2); other countries with fewer than two studies included: Lithuania, Ireland, Egypt, and United Kingdom [8,10,11,12,13,14,15,16,17,18,19,20,21,22,23,24,25,26,27,28,29,30,31,32,33,34,35,36,37,38,39,40,41]. The majority focused on experimental animal models (78%, n = 25). Only eight studies provided an analysis of human subjects [36,37,38,39,40,41]. We further divided the synthesis of the results into the following thematic categories: (1) Types of Tissue stem Cell Sources and Transplantation Strategies; (2) Mechanisms of Action and Cellular Effects; (3) Functional and Behavioural Outcomes in PD Models; (4) Immune, Inflammatory, and Microbiome Modulation; and, (5) Safety and Feasibility.

This analysis of studies on the application of tissue stem cells as a potential curative resource for Parkinson’s disease spans a range of years, reflecting the advancements in research and technology over time. The earliest studies in the dataset date back to 2010 [14,16,30,37]. Over the following years, there has been a noticeable increase in the number of studies, with more contributions around 2015 [8,10,15,31,36]. The trend continued with studies published around 2017 [11,27,28,33,41]. More recent studies, conducted around 2021, highlight the ongoing interest and development in this field [12,13,17,18,19,20,21,22,23,24,25,26,29,32,34,35,38,39,40]. This progression illustrates a growing body of knowledge and evolving methodologies aimed at harnessing the potential of tissue stem cells for treating Parkinson’s disease.

### 3.4. Types of Tissue Stem Cell Sources and Transplantation Strategies

Tissue stem cell therapies can involve either autologous or allogeneic transplantation strategies, depending on whether the donor and recipient are genetically identical (autologous) or not (allogeneic) within the same species. This classification applies to both animal and human studies and is critical for understanding immunological compatibility, safety, and therapeutic outcomes.

#### 3.4.1. Autologous Tissue Stem Cells

Autologous tissue stem cells are harvested from the individual’s own body, typically from blood or bone marrow, and reinfused. This approach minimises the risk of immune rejection since the cells are genetically identical to the recipient [13,19,21,41]. In tissue stem cell therapy for treating PD, autologous tissue stem cells, derived either from animal models or the patient’s own tissue, have been used to mitigate the risk of immune rejection and address ethical dilemmas. In PD studies, different sources of autologous tissue stem cells include bone marrow, adipose tissue, and induced pluripotent cells (iPSCs); each source offers unique advantages, and the extraction and usage methodologies vary from study to study [8,10,11,12,13,14,15,16,17,18,19,20,21,22,23,24,25,26,27,28,29,30,31,32,33,34,35,36,37,38,39,40,41]. For instance, one study described a protocol for isolating haematopoietic and mesenchymal stem cells from human bone marrow aspirated from the iliac crest; the bone marrow was processed using flow cytometry markers CD34+ and CD38– for haematopoietic stem cells and CD45– and CD146+ for mesenchymal stem cells [41]. This study reported improvements in PD symptoms such as motor function and quality of life, demonstrating the clinical relevance of bone marrow-derived stem cells in PD [41].

Adipose tissue also serves as a rich source of autologous tissue stem cells. Lian et al. outlined the isolation of human adipose-derived stem cells (hADSCs) from the inner thigh. The cells were obtained using type I collagenase digestion and cultured in a specific medium to confirm their multipotency through adipogenic and osteogenic differentiation assays [13]. Another group also reported the use of autologous ADSCs, where the cells were administered intravenously; despite the absence of a control group, improvements noted in patient assessments suggested the potential of ADSCs in treating PD [39]. Kim et al. took this a step further by labelling hADSCs with magnetic nanoparticles, which permitted the tracking of tissue stem cells; they saw that these cells were predominantly located in the substantia nigra, a critical area affected in PD [21]. Furthermore, mice with induced PD that received magnetic nanoparticle (MNP)-labelled hADSCs exhibited significant improvements in motor functions, with enhanced performance in apomorphine-induced rotation tests and rotarod tests, particularly noticeable six weeks after transplantation [21].

Induced pluripotent stem cells (iPSCs) also offer another promising avenue. iPSC lines have been developed in rhesus monkeys for both autologous and allogeneic transplantation [19]. These iPSCs differentiated into midbrain dopaminergic neural progenitors, essential for treating PD [21]. The study demonstrated a high expression of pluripotency markers and effective differentiation, highlighting the promise of iPSCs in personalised regenerative medicine.

The studies reviewed here emphasise the versatility and therapeutic promise of autologous tissue stem cells. These types of transplants have shown promising results in treating neurodegenerative diseases, particularly PD. Studies that utilised autologous tissue stem cells in animal models of PD demonstrated their potential to alleviate the symptoms of the disease and accurately target an important hotspot of the disease (the substantia nigra) [21,27]. Furthermore, human studies using autologous tissue stem cells have also shown promise, with important improvements in motor function and quality of life post-transplantation [13,39,41]. Consistent protocols for isolating and characterising autologous tissue stem cells are crucial for ensuring reproducibility and reliability; by standardising these processes, researchers can better compare results across studies and advance the field of autologous tissue stem cell therapy for PD.

#### 3.4.2. Allogeneic Tissue Stem Cells

Allogeneic tissue stem cells, as opposed to autologous tissue stem cells, are obtained from a donor, either genetically related or unrelated, and transplanted into a subject. While this method can introduce beneficial immune effects, it carries risks such as potential immune incompatibility and reactions [15,25,26,34,37,38]. Allogeneic tissue stem cell transplant also holds promise for treating PD, with marrow-derived stems cells as one of the most common cells used in humans [37,38]. Two studies in our review assessed the use of allogenic tissue stem cells from healthy donors in PD participants. One of the studies assessed the safety and tolerability of intravenous bone marrow-derived allogeneic human mesenchymal stem cells (MSCs), with a focus on anti-inflammatory effects and motor improvement post-infusion [38]; the other focused on human and bone marrow-derived mesenchymal stem cells (BM-MSCs) and evaluated their safety and efficacy when transplanted to the subventricular zone [37]. In both studies there was an important reduction in the Unified Parkinson’s Disease Rating Scale (UPDRS), and participants subjectively reported clarity in speech and a reduction in tremors and rigidity at 12 months of follow-up [37,38].

As for the animal models, one study used human bone marrow MSCs in a PD model of adult male Sprague–Dawley rats; they reported enhanced neuronal connections and neurite outgrowth after transplantation [25]. Other animal studies have also isolated MSCs from the bone marrow of C57BL/6 and Wistar mice and then administered them intranasally and by intracarotid infusion, respectively [15,27]. Others have used the C57BL/6 model to transplant bone marrow-derived neural stem cells (BM-NSCs) from green fluorescent protein (GFP) transgenic mice, which showed inhibitory effects in inflammation and M2 microglia polarisation [34]. Alternative transplantations sources have also been used in animal models. For instance, umbilical cord-derived MSCs administered intranasally have highlighted the neuroprotection and modulation of the brain–gut axis [26]. Additionally, human exfoliated deciduous teeth-derived stem cells (SHED) [12] and allogenic tissue stem cells from human buccal fat pad tissue (hBFP-ASC) [33] have also been used.

Other studies have focused on transplanting embryonic and neonatal stem cells from rats and humans. Researchers have used neural stem cells from neonatal Sprague–Dawley rats and primary foetal mouse tissue from C57/Bl6 female mice [28,29] or neural stem cells (NSCs) derived from late first-trimester human foetal cadavers and transplanted to mouse models to demonstrate their influence on mitochondrial bioenergetics and neuroprotection [20]. Other types of human cells used in animal models were human embryonic stem cells (ESCs), iPSC lines, and MSCs from human conjunctiva [12,32,35]. Certainly, various tissues sources have been used in allogenic tissue stem cell research (bone marrow, umbilical cord, dental pulp, adipose tissue, foetal tissue, etc), showing promising therapeutic outcomes. However, more standardised research is required to optimise protocols and understand the mechanisms of action and benefit of the different types of allogenic tissue stem cells in PD.

### 3.5. Mechanisms of Action and Cellular Effects

#### 3.5.1. Models of Dopaminergic Neuronal Loss

The mechanisms underlying dopaminergic neuronal degeneration in PD have been extensively investigated, with particular emphasis on the contribution of oxidative stress and mitochondrial dysfunction. The neurotoxin 6-hydroxydopamine (6-OHDA), a hydroxylated analogue of dopamine, was used by Lian and collaborators to induce a PD-like pathology in experimental models via direct injection into the substantia nigra, striatum, or medial forebrain bundle [13]. This approach mimics key features of PD, including oxidative damage, mitochondrial impairment, apoptosis, and neuroinflammation, culminating in marked dopaminergic cell loss [13]. Supporting this, another group reported that 6-OHDA undergoes rapid oxidation, generating reactive oxygen species (ROS) that further exacerbate neuronal demise in their animal models through mitochondrial failure and redox imbalance [25].

In terms of anatomical impact, 6-OHDA’s administration leads to pronounced dopaminergic degeneration within the striatum and the ipsilateral ventral tegmental area and substantia nigra pars compacta (SNc) [13]. A single intrastriatal injection is capable of eliciting a 60–70% loss of dopaminergic cell bodies in the SNc and 50–70% attrition of striatal terminals, thereby modelling the early symptomatic stages of PD [16]. Complementary findings in non-human primates using PET imaging with 11C-labelled dihydrotetrabenazine (DTBZ) revealed severe, unilateral reductions in DTBZ binding potential within the caudate and putamen, persisting over a 5–6-year period; furthermore, histological verification confirmed a greater than 80% depletion of TH-positive neurons in the substantia nigra [19].

Regarding therapeutic strategies, 6-OHDA-lesioned hemiparkinsonian rats have been utilised to evaluate the neurorestorative potential of MSC transplantation and the adenovirus-mediated delivery of glial cell-derived neurotrophic factor (GDNF) [25]. These interventions demonstrated neuroprotective effects, albeit contingent upon the preservation of a substantial portion of the nigrostriatal circuitry [25]. Moloney et al. similarly emphasised that the efficacy of GDNF’s delivery is predicated on the integrity of residual dopaminergic projections, highlighting the need for lesion models that emulate partial rather than complete degeneration [16]. In an alternative paradigm, systemic administration of the tissue stem cell-conditioned medium, SHED-CM, significantly ameliorated motor deficits and mitigated dopaminergic neuron loss in a rotenone-induced PD model [23].

Collectively, the use of 6-OHDA has been instrumental in modelling dopaminergic neurodegeneration via well-characterised pathways involving oxidative stress and mitochondrial dysfunction [13,16,25]. Parallel studies employing non-human primates have recapitulated comparable patterns of nigrostriatal degeneration, thereby validating 6-OHDA-induced models as reliable preclinical tools for dissecting PD’s pathogenesis and testing candidate therapeutics [19].

#### 3.5.2. Effects on Tyrosine Hydroxylase

Tyrosine hydroxylase (TH) is a useful biomarker to detect dopaminergic differentiation; however, it also acts as an enzyme essential for dopamine biosynthesis, catalysing the conversion of tyrosine into levodopa, the immediate precursor of dopamine. In PD, the degeneration of dopaminergic neurons in the substantia nigra leads to reduced dopamine levels, which underlies the characteristic motor symptoms; therefore, the expression and activity of TH serve as critical indicators of dopaminergic neuronal integrity, and a decrease in TH immunoreactivity is widely interpreted as a marker of neuronal dysfunction or loss [20,27,30]. Numerous studies in murine models of PD have demonstrated significant reductions in TH levels. For instance, a study using 1-methyl-4-phenylpyridinium (MPP+)-induced mouse models observed the progressive degeneration of TH-positive neuronal fibres through immunofluorescence [30]. Similarly, Pereira et al. reported cytoplasmic retraction and the loss of TH-positive synaptic terminals following 1-methyl-4-phenyl-1,2,3,6-tetrahydropyridine (MPTP)’s administration, further highlighting the vulnerability of these neurons to oxidative stress and neurotoxic insult [20].

Several investigations have examined the neuroprotective potential of tissue stem cell-based interventions on TH-positive neurons. Nesti et al. found that dental pulp stem cells (DPSCs) exerted a modest protective effect, evidenced by the presence of longer and more elaborate neurites in mesencephalic dopaminergic neurons [30]. In another study, the transplantation of hNSCs in PD models led to the reduced degeneration of TH-positive neurons within one week post-grafting; this was accompanied by measurable improvements in motor function, as assessed by behavioural testing [20]. Quantitative analyses further support these outcomes. Salama et al. assessed TH-positive cell populations in the SNc and the corpus striatum following rotenone treatment; dopaminergic neuron numbers in the SNc declined by 37 ± 8% relative to the control, while striatal fibre density was reduced to 53 ± 7% [27]. Notably, animals that received an intranasal administration of MSCs showed substantial neuroprotection: TH-positive cell counts in the SNc reached 80 ± 6%, and fibre density in the corpus striatum recovered to 91 ± 5% of control levels [27].

Further insight was provided by Qi et al., who evaluated the effect of LiCl-treated NSCs on the ventral tegmental area (VTA) of PD model rats; the proportion of TH-positive neurons was 51 ± 9% in the LiCl-treated group, compared with 46 ± 5% in the untreated group, indicating a modest benefit of pre-conditioning strategies [28]. Meanwhile, another study demonstrated the efficacy of GDNF-transduced MSCs in restoring dopaminergic function; despite the lesion-induced depletion in overall TH immunoreactivity, treated animals exhibited small but dense clusters of TH-positive staining within the striatum (findings absent in rats receiving unmodified MSCs) [16]. This localised immunopositivity likely reflected the sprouting of residual dopaminergic terminals towards the graft site, attracted by the high local concentrations of GDNF [16]. These results underscore the capacity of neurotrophic factors to create a microenvironment conducive to neuronal preservation and regeneration.

However, not all investigations reported a therapeutic benefit. Cerri et al. found that an intracarotid infusion of MSCs failed to prevent 6-OHDA-induced neurodegeneration; there were no significant changes in striatal TH-positive terminal density, nor in overall cytoarchitecture or neuronal morphology, when comparing treated and untreated animals [15]. Similarly, another group reported no significant differences between transplanted groups in terms of TH-positive cell number or volume [29]. Moreover, the projection distance of TH-positive fibres from the grafts remained unchanged, and no new striatal innervation was observed [29]. These findings highlight the variability in therapeutic efficacy across different experimental designs and cell sources and reinforce the importance of optimising both the cell phenotype and delivery strategy in future translational applications.

#### 3.5.3. Anti-Apoptotic Effects

The inhibition of apoptosis represents a critical therapeutic target for preserving dopaminergic neuronal populations. Several experimental strategies have focused on modulating apoptotic pathways to promote cell survival and attenuate the progression of disease. Lian et al. investigated the neuroprotective role of Pentraxin 3 (PTX3), a key protein involved in the innate immune response, demonstrating that it was associated with reduced neuronal apoptosis in a manner comparable to the protective effects observed with hADSCs [13]. Similarly, another study reported that the co-administration of Fasudil (a selective Rho-associated protein kinase (ROCK) inhibitor) and bone marrow-derived neural stem cells led to the upregulation of anti-apoptotic markers, thereby decreasing dopaminergic vulnerability in PD models [34]. In another complementary approach, researchers demonstrated that exosomes derived from SHED cells conferred neuroprotection by mitigating apoptosis in dopaminergic neurons exposed to PD-inducing toxins [23]. The mechanisms underlying this effect were attributed to the transfer of regulatory molecules capable of modulating the intracellular signalling pathways associated with cell survival.

Collectively, these findings showcase the potential of targeting apoptotic cascades in PD, whether through the action of specific proteins such as PTX3, pharmacological modulation via ROCK inhibition, or the deployment of tissue stem cell-derived exosomal products. Such strategies offer promising avenues for the development of neuroprotective interventions aimed at halting dopaminergic degeneration and altering the trajectory of disease progression.

#### 3.5.4. Neurotrophic Factors Induced by Tissue Stem Cells

Recent research has underscored the critical interplay between neurotrophic factors and the pathophysiology of PD, revealing that deficits in these molecules contribute significantly to neuronal susceptibility and progressive degeneration; furthermore, the contribution of MSC-derived factors to neuroregeneration and neuroprotection has been highlighted by multiple studies [16,20,22,24,30,37]. Neurotrophins are pivotal regulators of neural development and functional maintenance; they oversee a spectrum of processes including neurogenesis, neuroprotection, survival, and differentiation. These molecules influence cellular fate, promote axonal elongation, refine dendritic morphology, orchestrate synaptic innervation, and facilitate the synthesis of essential proteins necessary for sustained neuronal activity [30,37].

To contextualise these advances, foundational work laid the groundwork for understanding the neurotrophic capacity of MSCs. Moloney et al. demonstrated that MSCs are capable of secreting glial cell-derived neurotrophic factor (GDNF) and further, that this secretion can be enhanced via genetic modification to improve their neuroprotective efficacy [16]. Around the same period, Venkataramana et al. emphasised the role of neurotrophic factors released by bone marrow-derived MSCs (BMMSCs) in promoting neurogenesis and neuronal preservation within PD models, thereby establishing an early framework for tissue stem cell-based paracrine therapeutics [37]. Further expanding the molecular repertoire of MSC-derived secretomes, Chen et al. identified insulin-like growth factor binding protein-6 (IGFBP-6) and tissue inhibitor of metalloproteinases-2 (TIMP-2) as prominent components within the conditioned medium of dental pulp-derived stem cells [23]. These molecules exhibited both neuroprotective and neuroregenerative activity, underscoring the complexity and therapeutic versatility of tissue stem cell-derived secretomes in the PD context.

A recurrent limitation in tissue stem cell transplantation strategies for PD arises from the hostile neural microenvironment of the diseased brain, which is often depleted of supportive trophic signals and concurrently enriched with cytotoxic mediators. Despite this, various tissue stem cell types (such as induced pluripotent stem cells (iPSCs), neural stem cells (NSCs), and MSCs) exert therapeutic effects not solely through direct cellular replacement but via paracrine signalling [16,20,22,24,30,37]. These cells secrete neuroprotective factors and modulate the immune milieu, in part by suppressing neuroinflammatory pathways [16,20,22,24,30,37]. Particularly, GDNF secreted by mouse embryonic stem cells (mESCs) has demonstrated efficacy in supporting dopaminergic neurons’ survival and promoting their maturation [24]. In parallel, tissue stem cell populations such as dental pulp stem cells have been shown to secrete various types of neurotrophins, including brain-derived neurotrophic factor (BDNF), nerve growth factor (NGF), and GDNF, which exert targeted actions within the central nervous system, notably on motor neurons and the dopaminergic population of the substantia nigra [30].

Further evidence from a study by Park and Chang demonstrated that the intravenous administration of hASCs in MPTP-induced PD model mice successfully restored neurotrophic factor levels at dopaminergic terminals [22]. This restoration conferred a marked neuroprotection against oxidative stress, excitotoxicity, and apoptosis, while an enhanced expression of BDNF and GDNF translated into measurable improvements in motor performance [22]. Although outside the PD context, similar mechanisms have been observed in spinal cord injury models, wherein NSCs contributed to axonal regeneration and functional recovery via neurotrophin secretion [20]. In a complementary investigation, Moloney et al. reported that GDNF-expressing MSCs induced localised neurotrophic effects within the denervated striatum, evidenced by robust axonal sprouting from surviving dopaminergic terminals towards the transplant site [16]. A quantitative TH immunohistochemistry confirmed this sprouting response; however, despite the observed trophic benefit, full reinnervation of the striatum was not achieved, indicating that while such interventions are biologically active, further refinement is necessary to optimise their reparative capacity [16].

Together, these findings illustrate the diverse array of bioactive molecules secreted by MSCs and related tissue stem cell types, which collectively modulate key mechanisms of neurodegeneration and repair stimulating the local release of neurotrophic factors. The capacity of these factors to promote neuronal survival, suppress inflammation, and stimulate endogenous regeneration reinforces the therapeutic promise of MSC-based strategies in PD and merits continued translational exploration.

#### 3.5.5. Influence in Neuronal Differentiation

Following cell transplantation, researchers investigated whether the grafted cells could first differentiate into neurons and subsequently adopt a dopaminergic phenotype. Multiple strategies were employed to assess neuronal differentiation, each incorporating distinct experimental methods; for instance, Takahashi et al. used electron microscopy to analyse cellular morphology post-induction to reveal neuron-like structures that were supported by the expression of neural and neuron-specific markers [33].

To validate or induce neuronal differentiation, specific neuronal markers and associated antibodies have been utilised. One study identified the expression of microtubule-associated protein 2 (MAP2), homeobox 9 (HB9), and choline acetyltransferase (ChAT); these are definitive markers of motor neurons [21]. Another study applied lithium chloride (LiCl) treatment, which significantly increased the proportion of MAP2-positive cells (indicating neuronal maturation) while concurrently reducing the population of GFAP-positive glial cells [28]. Yang et al. employed a defined set of inductive factors with established roles in neurogenesis and differentiation: namely, SHH, GDNF, FGF8α, and TGFβ3 [17]. They reported neuron-like morphological changes within one to four weeks of induction; these findings further support the feasibility of guided neuronal differentiation using precise molecular cues [17].

#### 3.5.6. Influence in Dopaminergic Differentiation

TH is a molecular marker expressed in neurons and endocrine cells, including dopaminergic and noradrenergic populations; its presence makes it essential for identifying dopaminergic differentiation within the central nervous system [35]. Early studies focused on detecting dopaminergic cells post-transplantation and confirmed both their differentiation and spontaneous electrophysiological activity. Notably, human neuroepithelial cells derived from induced pluripotent tissue stem cells displayed these properties. The therapeutic potential of conjunctiva-derived MSCs was also explored; once differentiated into dopaminergic neurons, they managed to increase dopamine expression in the striatum [35].

Subsequent research demonstrated that transplanted dopaminergic precursors could mature into neurons expressing TH, along with other neuronal markers such as βIII-tubulin, NF200, MAP2, and GFAP; these findings highlighted the promising role of hBFP-ASCs in PD therapies [29,33]. In parallel, pan-neuronal markers (Tuj-1, DAT, and Nurr1) were also detected in TH-positive cells; interestingly, these markers were absent in control groups three weeks after induction [17]. TH-positive fibre innervation in the striatum was further assessed using image analysis techniques, contributing to the anatomical validation of the graft’s efficacy [27].

Efforts to enhance dopaminergic differentiation included the use of specialised culture media. When human adipose-derived stem cells (HADSCs) were placed in a neurobasal medium and exposed to an induction cocktail for 12 days, increased dopamine production was observed; this was accompanied by the formation of neurospheres [28]. Other approaches have also emerged; for example, lithium chloride was employed to stimulate neural stem cell (NSC) proliferation both in vitro and in PD animal models following transplantation [28]. In another strategy, mouse embryonic stem (ES) cells (R1) were infected with LvNurr1 and LvGPX-1 to generate cells co-expressing Nurr1 and GPX-1; these were subsequently differentiated into dopaminergic neuron-like cells using a three-dimensional PCL/Mtg (Poly-ε-Caprolactone/Matrigel) scaffold [31].

Multiple molecular pathways have been implicated in dopaminergic differentiation. Yan et al. described the activation of the Wnt signalling pathway to promote neuronal proliferation, alongside PI3K/Akt/mTOR signalling for neuroprotection; both pathways appeared to synergise in safeguarding dopaminergic neurons [34]. Likewise, Kriks et al. underscored the relevance of Wnt signalling via CHIR99021 exposure, which supports roof plate specification and midbrain dopaminergic neuron development [14].

#### 3.5.7. Effects on Electrophysiological Activity

The electrophysiological behaviour of dopaminergic neurons, both in vitro and in vivo, is marked by distinctive and reproducible activity patterns integral to their physiological function. In one study employing time-series calcium imaging in differentiated neuronal cultures, spontaneous activity was detected, characterised by action potentials propagating along neuritic processes [14]. These neurons exhibited regular spontaneous calcium transients underpinned by rhythmic spiking, consistent with earlier reports describing the tonic firing properties of dopaminergic neurons in vitro; particularly, dopaminergic neurons identified by TUBβIII and TH immunoreactivity displayed a more regular firing cadence in comparison with their TH-negative counterparts, supporting their electrophysiological resemblance to nigrostriatal dopaminergic neurons observed in vivo [14]. In contrast, TH-negative cells demonstrated a more erratic depolarisation profile, further reinforcing the specificity of the observed phenotype to dopaminergic identity [14].

These findings are corroborated by in vivo characterisations of SNc dopaminergic neurons, which exhibit a slow, spontaneous firing rate between 1 and 3 Hz, accompanied by sub-threshold oscillatory potentials [14]. Importantly, such electrophysiological hallmarks were preserved in SNc dopaminergic neurons cultured from early postnatal murine tissue after 2–3 weeks in vitro. Furthermore, dopaminergic neurons derived from embryonic tissue stem cells via floor plate induction protocols recapitulated these cardinal electrophysiological features, affirming their maturation into a functional SNc-like phenotype and further affirming the potential of tissue stem cells to regenerate PD-related tissue [14].

Collectively, these studies highlight the conserved electrophysiological signature of tissue stem cell-derived dopaminergic neurons across experimental models. The observation of regular firing patterns and stereotypical oscillatory behaviour in both in vitro differentiated and native SNc neurons underscores their neurophysiological fidelity [14]. TH-positive neurons, in particular, demonstrated a firing profile reminiscent of mature nigrostriatal neurons, further validating their identity and maturity [14]. Crucially, the ability of ES cell-derived dopaminergic neurons to attain the electrophysiological phenotype characteristic of SNc neurons signifies successful functional differentiation, a prerequisite for their potential application in disease modelling and regenerative therapies.

#### 3.5.8. Results in α-Synuclein Modulation

Recent investigations have elucidated critical aspects of α-synuclein modulation in PD, with particular emphasis on its aggregation, intercellular propagation, utility in disease modelling, and relevance to therapeutic development. A hallmark of PD pathogenesis involves the propensity of α-synuclein to misfold and accumulate as intracellular aggregates, ultimately contributing to neuronal toxicity and degeneration [8]. Certainly, α-synuclein is actively secreted by neuronal cells via exocytosis, while both neurons and glial cells internalise extracellular α-synuclein aggregates through endocytic mechanisms [8].

From a therapeutic standpoint, passive immunisation strategies targeting α-synuclein have demonstrated considerable potential. Antibodies directed against oligomeric species appear capable of interrupting aggregation dynamics at the neuronal membrane, facilitating lysosomal degradation via autophagy and enhancing the clearance of extracellular deposits [8]. Complementary approaches have explored cell-free biologics; for instance, conditioned medium derived from tissue stem cells obtained from human exfoliated deciduous teeth (SHED) was shown to attenuate the α-synuclein burden within the striatum [23]. In a rotenone-induced PD model, the systemic administration of SHED significantly ameliorated motor deficits, elevated striatal TH levels, and reduced the α-synuclein pathology in both the nigrostriatal axis and substantia nigra [23].

Beyond its pathogenic role, α-synuclein serves as a critical substrate for experimental disease modelling. The combined delivery of adeno-associated virus (AAV)-encoded α-synuclein with exogenous fibrils has been used to induce an accelerated pathological cascade, culminating in progressive behavioural impairment and substantial dopaminergic neuronal loss [32]. Whilst transgenic murine models and viral-mediated α-synuclein overexpression systems have contributed to our understanding of PD, they are often constrained by a protracted onset and phenotypic variability. In contrast, humanised α-synuclein models more faithfully recapitulate core features of the disorder and afford an extended platform for evaluating long-term therapeutic efficacy, including regenerative strategies such as cell-replacement therapy [32].

Elucidating the molecular determinants governing α-synuclein’s aggregation and spread remains imperative for the rational design of targeted interventions in PD. Immunotherapeutic strategies and biologically active tissue stem cell-derived products, such as SHED, hold promise for both disease modification and symptomatic relief. Moreover, refined in vivo models offer valuable insights into the disease’s kinetics and therapeutic windows. Continued translational research will be essential to validate these emerging paradigms and facilitate their integration into clinical frameworks.

### 3.6. Functional and Behavioural Outcomes in PD Models

#### 3.6.1. General Therapeutic Benefits

To evaluate the efficacy of tissue stem cell-based therapies for PD, researchers have assessed not only symptomatic improvement but also cellular and molecular outcomes: specifically, the capacity of transplanted cells to promote dopaminergic differentiation, neuronal regeneration, and the restoration of damaged circuits. The transplantation of embryonic tissue stem cell-derived neural progenitors and mesenchymal tissue stem cell-derived neural progenitors via intravenous administration has been associated with sustained motor improvements and a marked reduction in reactive astroglial and microglial burden [18]. Similarly, hASCs were shown to rescue dopaminergic neurons and restore motor function, primarily through the upregulation of neurotrophic factors such as BDNF and GDNF [21,22].

While therapeutic benefits were anticipated, it was also essential to evaluate the broader systemic effects of tissue stem cell transplantation, both beneficial and adverse. In terms of safety, both autologous and allogeneic transplantation modalities were generally well tolerated; however, successful engraftment in allogeneic grafts was minimal or absent without proper immunosuppression [8,10,11,12,13,14,15,16,17,18,19,20,21,22,23,24,25,26,27,28,29,30,31,32,33,34,35,36,37,38,39,40,41]. By contrast, autologous transplantation was not only immunologically favourable but also associated with improved behavioural profiles—most notably, a reduction in depressive symptoms—positioning it as a promising avenue for adjunctive therapy in neuropsychiatric manifestations of PD [19].

Neuroinflammation has also emerged as a critical therapeutic target. The systemic administration of tissue stem cell-derived conditioned medium, particularly that from the tissue stem cells of human exfoliated deciduous teeth, significantly reduced Iba1-positive microglia and upregulated genes involved in neurodevelopment and axonal regeneration; these changes collectively contributed to a measurable attenuation of neuroinflammatory responses in PD animal models [23]. Another essential consideration is the durability of therapeutic effects following transplantation. In a longitudinal study, Tao et al. demonstrated that clinical rating scores in both autologous and allogeneic transplant recipients improved in the months following transplantation and remained stable over a 6–12 month follow-up period [19]. Embryonic stem cells (ESCs) and MSCs also produced motor benefits that persisted for at least three months, albeit with a diminished effect compared to the initial 10-day post-treatment window [18]. Interestingly, while human hBMSCs were still detectable near the substantia nigra 28 days after transplantation, pre-differentiated hBMSCs (i.e., those induced into neuron-like cells prior to grafting) were not observed at the injection site during the same post-transplant interval [25]. This observation suggests that undifferentiated or minimally pre-conditioned cells may possess superior engraftment potential in vivo, possibly due to enhanced adaptability within the host microenvironment.

#### 3.6.2. Motor Outcomes

The assessment of mobility-related outcomes is central to evaluating the therapeutic efficacy of tissue stem cell-based interventions in PD; multiple studies have reported improvements in motor performance across various PD animal models following treatment with tissue stem cells or their derivatives [12,13,18,19,20,21,26,27,28,37]. Narbute et al. demonstrated that the administration of extracellular vesicles derived from human dental pulp stem cells led to significant improvements in coordination and posture in post-lesion 6-OHDA rats [12]. Stand, stride length, and step cycle analyses all indicated a normalisation of gait patterns when compared to untreated lesion controls [12]. Supporting these findings, another group reported that hADSC treatment, in a similar model, accelerated movement velocity, increased locomotor distance, and reduced sedentary resting time, reflecting global improvements in motor activity [13]. Complementary evidence was provided by Pereira et al., who observed that human neural stem cell engraftment significantly ameliorated hypokinesia, catalepsy, and bradykinesia in MPTP-treated mice; the preservation of dopaminergic neurons correlated with a marked reduction in PD-like motor symptoms, highlighting a strong neuroprotective component to functional recovery [20]. Another study quantified the effect of tissue stem cell transplants by using positron emission tomography (PET) with dihydrotetrabenazine (DTBZ); they revealed significantly elevated binding potential values in the ipsilateral putamen and caudate nucleus of autologous transplant recipients [19]. These neuroimaging findings were closely associated with improvements in motor performance, suggesting a robust graft–host integration and dopaminergic reinnervation [19]. Complementarily, Precious et al. reported that mice receiving epiblast stem cell transplants exhibited a partial recovery in the rotarod test, with performance metrics comparable to or exceeding those achieved by animals receiving ventral mesencephalon grafts [29]. Additionally, Acquarone et al. demonstrated that the transplantation of mitomycin C (MMC)-treated mouse embryonic tissue stem cells led to significant gains in locomotor activity, coordination, and grip strength, restoring motor function to levels approaching those of non-lesioned controls [10]. Collectively, these findings underscore the therapeutic potential of diverse tissue stem cell-based strategies in restoring motor function and dopaminergic integrity in preclinical PD models.

Temporal analyses have revealed that motor improvements may vary according to the post-transplantation interval. In a study, rats receiving LiCl-preconditioned NSCs exhibited significantly reduced motor deficits at both 8- and 12-weeks post-transplantation, with reductions in motor impairment reaching 53 ± 9% (*p* < 0.001) and 57 ± 7% (*p* < 0.001), respectively, when compared with sham-operated controls [28]. Similarly, Kim and Chang used the rotarod test to assess coordination and balance following hASC transplantation; significant reductions in contralateral net turning were observed on day 33 in the 6-OHDA/ASC-MNP group, although no significant change was detected on day 12, indicating a delayed therapeutic onset [21]. Another study of rat models receiving umbilical cord MSCs reported a shortened descent time in the pole test (6.11 ± 0.16) and higher traction test scores (3.08 ± 0.23), suggesting enhanced muscle strength and coordination [26]. Further analysis of the rat’s gut microbiota revealed several taxa that were negatively correlated with motor performance; specifically, Gammaproteobacteria, Enterobacteriaceae, Lactobacillaceae, Enterobacteriales, Lactobacillales, Escherichia-Shigella, Alistipes, Lachnoclostridium, and Prevotella 9 were all inversely associated with traction test scores [26]. Conversely, the presence of Proteobacteria, Gammaproteobacteria, Enterobacteriaceae, Lactobacillaceae, Enterobacterales, Lactobacillales, Escherichia-Shigella, and Prevotella 9 showed a positive correlation with a prolonged pole descent time, further supporting their link to motor dysfunction [26]. These findings underscore the multifaceted nature of PD pathology, suggesting that the gut microbiota’s composition may influence motor outcomes through neuroimmune and metabolic pathways. The observed improvement following UC-MSC treatment not only highlights its potential in alleviating motor deficits but also suggests an ancillary role in modulating gut–brain axis dysregulation.

Long-term outcomes were further explored by Tao et al., who compared autologous versus allogeneic tissue stem cell transplantation in monkeys. Autologous graft recipients showed an enhanced movement frequency, increased locomotor speed, and improved motor fluidity, with clinical improvements initiating a few months after treatment and stabilising between 6 and 12 months [19]. The average recovery ratio was approximately 40%, with some subjects reaching 60%; fine motor assessments revealed restored use of the ipsilateral hand, a result that persisted across both graft types, though more prominently in the autologous group [19]. Furthermore, sustained improvements in motor function, including movement, speed, and fine motor control, were seen up to 24 months post-autologous tissue stem cell transplantation, with specific mention of improvements in symptoms such as tremor and rigidity and enhanced well-being [19]. Another study looking into MPTP-treated mice found that the repeated intravenous administration of hASCs led to significant reductions in latency time during the rotarod test [22]. Prior to testing, mice were conditioned to 5 rpm for five minutes; the following day, they were evaluated at an accelerated speed (5 to 40 rpm over 10 min). The hASC-treated group demonstrated improved motor endurance and coordination, suggesting the recovery of dopaminergic neurons and associated transporter systems [22]. In contrast, Salama et al. offered a multidimensional behavioural analysis in rotenone-exposed mice. Following MSC administration, performance improvements were noted across four domains: akinesia, catalepsy, the open field test, and the parallel rod test. These findings provide robust behavioural evidence supporting the motor-restorative potential of MSCs [27].

Finally, improvements in motor outcomes have also been seen in human studies, with reported improvements in the Unified Parkinson’s Disease Rating Scale (UPDRS) [38,39,40]. One study reported an improvement in UPDRS (OFF medication) at 4 years of follow-up in six of seven participants [40]. Similarly, another study reported a 18–31% improvement with regards to motor disability in the UPDRS [37]. Schiess et al. reported an important improvement in the UPDRS motor score, as well as in the overall UPDRS score, in those patients that received the highest tissue stem cell dosage (10 × 10^6^ cells/kg) [38]. Finally, other authors reported a subjective motor improvement, such as improved speech, swallowing, dexterity, handwriting, walking stability, range of motion, and balance, as well as decreased tremors, bradykinesia, and rigidity [36,41]

However, despite these promising results, not all studies reported promising motor outcomes. For instance, Cerri et al. found that MSC infusion did not modify the progression of 6-OHDA-induced degeneration, nor did it impact motor performance in the stepping test [15]. Similarly, Moloney et al. reported that transplanted tissue stem cells failed to confer neuroprotection; treated animals developed lateralised motor impairments and exhibited a significant loss of TH+ neurons and terminals [16].

#### 3.6.3. Behavioural Outcomes

Behavioural recovery is a critical endpoint in assessing the therapeutic efficacy of tissue stem cell-based interventions; a range of preclinical and clinical studies have reported improvements in various behavioural domains, including motor coordination, exploratory activity, and general well-being [17,18,19,20,21,23,25,26,27,28,37].

Salama et al. demonstrated that the intracerebral administration of MSCs improved behavioural performance in PD models by enhancing general activity and reducing cataplexy [27]. In parallel, Lian et al. observed that mice treated with hADSCs exhibited accelerated locomotion, an increased travel distance, and a reduced resting time, suggesting restored vitality [13]. Similarly, another study showed that the transplantation of human neural stem cells one week after MPTP-induced injury reduced TH+ neuronal loss in the substantia nigra, which was associated with the amelioration of catalepsy [20]. Behavioural recovery was also noted in models treated with both embryonic tissue stem cells and MSCs, with improvements reported as early as 10 days post-transplantation [18]. Also, dosage seems to play a role in behavioural improvement, as shown by Chen et al., who evaluated conditioned medium from tissue stem cells of human exfoliated deciduous teeth, showing that only the 100 μg/mL dose elicited a significant behavioural improvement, whereas 10 μg/mL and 400 μg/mL did not show any measurable effect [23].

The autologous and allogeneic transplantation of dopaminergic neurons in monkey models were also compared by researchers, who showed that the autologous group exhibited more substantial behavioural gains, including greater purposeful movement and improved fluidity [19]. Furthermore, monkeys from the allogenic transplant group showed a lack of motivation and self-harm behaviour before and after transplantation; in contrast, none from the autologous group presented self-harm behaviour, and the only monkey that presented a lack of motivation before transplantation showed a complete remission after treatment [19]. These functional improvements were sustained over time, reinforcing the potential of autologous approaches for long-term behavioural benefits [19]. Positive effects were also reported using tissue stem cells from alternative sources. Yang et al. found that human spermatogonial stem cell transplantation led to behavioural recovery in striatal-lesioned mice [17]. In a separate study, Qi et al. demonstrated that the pre-treatment of NSCs with lithium chloride enhanced cognitive performance in PD models, evidenced by improved learning and memory capacity [28].

Clinical studies have echoed these findings from animal models. Venkataramana et al., using UPDRS, observed a 17.92% improvement during the “off” medication period and a 31.21% improvement during the “on” period, across domains such as mental status and daily functioning [37]. Additionally, studies have also reported subjective behavioural improvements, with patients reporting increased emotional well-being and motivation after treatment [37,41]

#### 3.6.4. Anti-Inflammatory Effects

Chronic neuroinflammation, characterised by persistent microglial activation and the release of pro-inflammatory cytokines, has been strongly implicated in the progression of neurodegenerative conditions, including PD. As a result, tissue stem cell-based interventions are increasingly being explored for their capacity to mitigate inflammation, limit neuronal injury, and promote functional recovery by restoring a more favourable neuroimmune environment [20,23,26,34]. In the healthy brain, microglia contribute to homeostasis and support neuronal development; however, when chronically activated, they become potent sources of neurotoxic mediators. In a PD mouse model induced by MPTP, researchers observed that serum levels of TNF-α, IL-6, and LPS were significantly elevated compared to controls [26]. However, following treatment with umbilical cord-derived mesenchymal stem cells, inflammatory responses were attenuated, with a marked reduction in pro-inflammatory status; this intervention also inhibited reactive gliosis and facilitated both locomotor improvement and functional motor recovery [42].

A complementary study by Yan et al. showed that combining Fasudil (a Rho-associated protein kinase inhibitor) with bone marrow-derived neural stem cells suppressed the expression of NLRP3, IL-1β, and TNF-α, while concurrently enhancing anti-inflammatory activity through increased IL-10 expression [34]. When administered separately, both agents independently reduced iNOS expression and elevated arginase-1 levels; however, their combination yielded amplified therapeutic effects [34]. In another model, Pereira et al. demonstrated that tissue stem cell transplantation could reduce the expression of genes associated not only with pro-inflammatory cytokines but also with T cell proliferation, thereby generating a local environment with reduced cytotoxicity and improved neuroprotection [20]. Further support for these anti-inflammatory effects has been shown when evaluating the impact of conditioned medium derived from the stem cells of human exfoliated deciduous teeth in a rotenone-induced PD rat model [23]. SHED-CM treatment upregulated genes involved in neurodevelopment and nerve regeneration and modulated several molecular networks associated with serotonergic and cholinergic synapses, calcium signalling, and axon guidance [23]. These molecular adaptations suggest an integrated mechanism that extends beyond the control of inflammation to encompass support for neuronal circuit repair.

Collectively, these studies underscore the capacity of tissue stem cell-based therapies to modulate chronic neuroinflammation and restore homeostatic balance. By targeting key inflammatory pathways and inducing regenerative gene expression profiles, such interventions hold promise for attenuating PD’s pathology and promoting functional recovery.

### 3.7. Safety of MSC Transplants

While tissue stem cell therapies hold substantial promise for PD, as discussed at length before, their pluripotent and proliferative properties raise concerns regarding the potential for formation of tumours. To assess this risk, several studies have examined both short- and long-term outcomes following the transplantation of various tissue stem cell types [10,18,22,29,33].

In one investigation, researchers evaluated neural progenitor (NP) grafts by means of a comprehensive histological analysis of the brain, lungs, spleen, kidney, and liver in treated animals [18]. No evidence of tumour formation or ectopic cell masses was detected, indicating a favourable safety profile for NP transplantation [18]. Similarly, human adipose-derived stem cells were found to be non-tumourigenic; no abnormal growths, chromosomal aberrations, or signs of immune rejection have been reported in any of the transplanted brains [22,33]. Conversely, mouse embryonic stem cells (mESCs) demonstrated a higher tumourigenic potential. Acquarone et al. reported the development of teratomas approximately 12 weeks after mESC transplantation. However, this adverse outcome was preventable: pre-incubation of the cells in vitro with 1 µg/mL mitomycin C (MMC) for 12 h effectively inhibited the formation of tumours, suggesting a viable approach to mitigate the associated risks [10].

Beyond initial tumour surveillance, long-term follow-up has also been critical in determining the duration of tumour-free outcomes. In the case of dopaminergic grafts derived from mouse epiblast stem cells, no signs of tumourigenesis were observed for at least 16 weeks post-transplantation [29]. Notably, MMC-treated mESCs maintained their therapeutic effect without any detectable tumour formation for up to 15 months, the longest duration reported to date. This was followed by NP-based therapies, which remained non-tumourigenic for a period of nine months [10,18]

These findings showcase the critical importance of both cell type selection and pre-treatment strategies in ensuring the safety of tissue stem cell-based interventions. With appropriate precautions, several tissue stem cell types (including NPs, hASCs, and MMC-preconditioned ESCs) can be employed without incurring tumourigenic risks, thereby enhancing their viability for clinical applications in PD if validated by future studies.

## 4. Discussion

Our scoping review consolidates and contextualises current knowledge on the application of tissue stem cell-based therapies in the treatment of PD, offering a panoramic view of developments across experimental models and clinical studies. Our findings underscore a steadily growing body of evidence supporting the efficacy of tissue stem cells; particularly mesenchymal stem cells (MSCs), neural stem cells (NSCs), and induced pluripotent stem cells (iPSCs), in ameliorating dopaminergic degeneration, modulating neuroinflammation, and improving functional outcomes in PD [8,10,11,12,13,14,15,16,17,18,19,20,21,22,23,24,25,26,27,28,29,30,31,32,33,34,35,36,37,38,39,40,41].

The evidence supports the notion that tissue stem cell therapies operate through multifaceted mechanisms. Beyond direct cell replacement, transplanted tissue stem cells consistently demonstrate paracrine activity, secreting a diverse array of neurotrophic and anti-inflammatory factors (including GDNF, BDNF, IGFBP-6, and TIMP-2) [16,20,22,24,30,37]. These molecules exert regulatory effects on the host microenvironment, thereby supporting dopaminergic neuron survival, promoting synaptic and axonal repair, and enhancing functional connectivity [16,20,22,24,30,37]. Furthermore, recent investigations into the gut–brain axis suggest that systemic immune modulation may constitute a significant therapeutic pathway. This perspective represents a conceptual shift, positioning tissue stem cell therapy as a systemic rather than a purely localised neurorestorative strategy [26]. Moreover, emerging areas of investigation, including the use of tissue stem cell-derived exosomes and the targeted modulation of α-synuclein pathology, further diversify the therapeutic landscape [30]. The demonstrated capacity of SHED-derived conditioned medium to reduce α-synuclein aggregation illustrates a promising cell-free strategy, with particular relevance for early-stage interventions or for patients contraindicated for cell transplantation. In addition to these benefits, we found that motor recovery was the most consistently reported outcome, often linked to the preservation or regeneration of tyrosine hydroxylase (TH)-positive neurons, restoration of dopaminergic synthesis, and striatal reinnervation [12,13,18,19,20,21,26,27,28,37]. However, the variability in behavioural outcomes, particularly in assessments such as the rotarod, pole test, and Unified Parkinson’s Disease Rating Scale (UPDRS), underscores the methodological heterogeneity across studies and calls for a greater standardisation in behavioural evaluation.

Our analysis also reveals a complex interplay between the source of tissue stem cells and their therapeutic efficacy. Autologous transplants, particularly those derived from adipose tissue or bone marrow, were associated with enhanced biocompatibility, minimal immune rejection, and sustained functional improvement in both human and preclinical models [13,19,21,41]. In contrast, allogeneic approaches, though logistically advantageous, exhibited more variable outcomes, often requiring immunosuppression, which may confound the interpretation of therapeutic efficacy and increase systemic risks; studies using iPSCs from non-human primates, notably rhesus macaques, provided compelling preclinical data by replicating both the disease pathology and personalised response to therapy [15,25,26,34,37,38].

Despite encouraging results in terms of motor and behavioural recovery, a subset of studies failed to demonstrate meaningful benefits [15,16]. Such discrepancies appear to reflect an interplay of factors, including the state of differentiation of transplanted cells, the timing and route of administration, and the condition of the host environment. For instance, delayed transplantation following extensive neuronal loss may limit the restorative potential of otherwise viable grafts. Similarly, insufficient pre-conditioning (of the host or the transplanted cells) may compromise engraftment, survival, or integration [10,18,22,29,33]. Additionally, safety remains a key consideration. While many tissue stem cell types were shown to be non-tumourigenic over extended follow-up periods, embryonic stem cells (particularly mESCs) retain a well-documented risk of teratoma formation unless appropriately pre-treated [10]. Notably, the use of mitomycin C has been shown to abrogate this risk, providing a tangible strategy to enhance clinical safety [10]. Nonetheless, long-term surveillance remains a pressing need; the potential for ectopic differentiation or functional drift post-transplantation is insufficiently studied, highlighting the value of advanced in vivo imaging and molecular tracking technologies.

Although promising preclinical evidence exists, the clinical translation of tissue stem cell therapies for PD remains in an early phase. As of now, only a limited number of clinical trials have tested tissue-derived stem cell interventions in PD patients, most of which are Phase I/II studies focused on safety and feasibility rather than efficacy. These include trials using autologous bone marrow-derived MSCs [37], allogeneic MSCs [38], and hESC-derived neural progenitors [40], which have demonstrated acceptable safety profiles and modest improvements in UPDRS scores. However, the translatability of preclinical findings to human patients is hindered by several challenges. Firstly, animal models such as 6-OHDA and MPTP-lesioned rodents do not fully replicate the progressive and multifactorial pathology of human PD. Secondly, the variability in stem cell sources, differentiation protocols, and delivery methods limits reproducibility and regulatory standardisation. Thirdly, the immune response in humans, especially to allogeneic or xenogeneic grafts, may differ substantially from that observed in animal models, necessitating immunosuppressive strategies that can complicate clinical applications. Additionally, clinical trials must address the long-term safety risks, such as ectopic growth or tumourigenicity, particularly when using pluripotent stem cells. Monitoring engraftment, cell fate, and functional integration remains technically challenging, underscoring the need for advanced in vivo imaging and tracking technologies.

To advance clinical applications, future research must prioritise the following: (1) the development of standardised protocols for cell isolation and differentiation; (2) rigorous, multicentre trials with harmonised outcome measures; (3) biomarkers to predict response and guide patient selection; and (4) a longitudinal follow-up to assess sustained efficacy and safety. Collaborative efforts involving neuroscientists, clinicians, and regulatory agencies will be crucial to translating experimental promise into clinically viable therapies for PD.

In summation, our synthesis highlights that tissue stem cell therapy for PD constitutes a dynamic platform encompassing multiple, interacting therapeutic approaches. As this field moves closer to clinical applications, several priorities emerge: the optimisation and standardisation of protocols for cell isolation and differentiation; development of minimally invasive and targeted delivery systems; rigorous, long-term safety and efficacy monitoring; and identification of patient-specific biomarkers predictive of therapeutic response. By tackling these matters, tissue stem cell-based therapies will become a promising, multifactorial intervention with the potential to alter the trajectory of Parkinson’s disease and improve patients’ quality of life. Realising this potential will require multidisciplinary collaboration, sustained regulatory oversight, and continued innovation across the intersecting domains of cell biology, translational neuroscience, and clinical trial design.

### 4.1. Ongoing Controversies and Ethical Considerations

Despite encouraging findings, the field of tissue stem cell therapy for PD remains troubled with important scientific and ethical challenges. A major concern lies in the risk of premature clinical translation without robust regulatory oversight or the standardisation of protocols. As highlighted in multiple studies, differences in cell isolation methods, delivery strategies, and differentiation protocols contribute to a wide heterogeneity in outcomes. Without a standardisation of these techniques, direct comparisons are limited, and reproducibility is compromised. Furthermore, the use of pluripotent stem cells, particularly embryonic stem cells, raises concerns about tumourigenicity, as documented in certain preclinical models [10]. Although mitigation strategies such as mitomycin C pre-treatment have demonstrated success in reducing this risk, these techniques are not yet universally applied. Similarly, immunological incompatibility in allogeneic grafts necessitates a careful evaluation of the risk of rejection and the long-term effects of immunosuppressive regimens. Ethically, the application of stem cell interventions must be guided by caution. Several studies have reported functional benefits in small patient cohorts; however, these findings remain exploratory and lack the statistical power to support clinical adoption. We caution against drawing therapeutic conclusions from early-phase trials or preclinical data, and strongly endorse the position that stem cell therapy in PD must be pursued within well-regulated, rigorously monitored clinical research frameworks. Broader consensus from the scientific and regulatory communities is essential before these interventions can be translated into routine clinical use.

### 4.2. Limitations

Our scoping review has some limitations. First, although we captured a broad range of studies, the variability in experimental designs, animal models, cell types, and outcome measures limited direct comparisons across studies; the diverse nature of the experimental designs, ranging from in vitro cellular assays to animal models and early-phase human trials, limits the direct comparability of outcomes, and the differences in species, stem cell types (e.g., MSCs, iPSCs, NSCs), methods of cell delivery, dosing regimens, timing, and behavioural endpoints further complicate data synthesis. Similarly, in vitro studies may provide mechanistic insights without necessarily predicting in vivo efficacy. While scoping reviews aim to map the breadth of the available evidence, this heterogeneity underscores the need for caution when extrapolating results across models or drawing generalised conclusions about clinical applicability. Future research should prioritise harmonised protocols and standardised outcome measures to enhance comparability and translational value. Second, most of the included evidence is derived from preclinical research, and only a minority of studies involved human participants, thus restricting the clinical generalisability. Third, many studies lacked long-term follow-up data, impeding our ability to assess their sustained efficacy and late-onset adverse effects, including tumourigenesis (especially in humans). Fourth, we did not assess risk of bias or methodological quality formally, as this is beyond the scope of a scoping review. Lastly, publication bias may have influenced our findings, as studies with null or negative results may be under-represented in the literature, especially when related to translational and experimental endeavours. It is critical to emphasise that our review does not support any clinical recommendations. The current evidence is largely preclinical and exploratory, and there remains no standardised or validated therapeutic protocol for stem cells’ application in PD. Although we have identified promising cellular mechanisms and observed potential benefits in animal and early-stage human models, these findings should be interpreted with caution. A further clinical validation, standardisation, and safety evaluation are required before therapeutic conclusions can be drawn or clinical adoption considered.

## 5. Conclusions

Tissue stem cell therapies represent a multifaceted and promising approach to the treatment of Parkinson’s disease, operating through a range of mechanisms including neurotrophic support and anti-inflammatory and anti-apoptotic effects, as well as the modulation of α-synuclein aggregation and the gut–brain axis. Autologous transplants have shown superior safety and efficacy profiles compared to allogeneic sources, with consistent improvements observed in both motor and behavioural outcomes across preclinical and early clinical studies. Nevertheless, clinical translation remains constrained by methodological heterogeneity and persisting safety concerns, particularly those related to tumourigenicity and heterogeneous results. The development and implementation of standardised protocols for cell preparation, delivery, and outcome assessments will be critical to advancing the field, which we believe holds significant potential to alter the course of the disease. We advise against deriving therapeutic claims from early or preclinical studies and emphasise that stem cell therapies for PD should be developed strictly within regulated, closely monitored clinical research frameworks.

## Figures and Tables

**Table 1:** Relevant characteristics of the included studies stratified by type of subject.

Reference	Country	Year	Study Design	Type of Subject	Source of Tissue Stem Cell
Oh, Se Hee [8]	South Korea	2016	Experimental	In vitro and male C57BL/8 mice	Humans
Acquarone, Mariana [10]	Brazil	2015	Experimental	Mouse	Mouse’s mESCS
Calice, Caroline [11]	Brazil	2017	Experimental	Mice	Bone marrow mononuclear cells (BMMCs)
Narbute, Karīna [12]	Lithuania	2019	Experimental	Male Wistar rats	Human exfoliated deciduous teeth of a child
Lian, Changlin [13]	China	2021	Experimental	Male mice	Human sterile adipose tissue obtained from the fat of the inner left thigh
Kriks, Sonja [14]	United States	2011	Experimental	Adult rodent + two adult rhesus monkeys	Human engraftable midbrain dopaminergic neurons
Cerri, Silvia [15]	Italy	2015	Experimental	Male Wistar rats	MSCs isolated from bone marrow stromal cells collected from femurs and tibias of male Wistar rats 6–8 weeks old
Moloney, Teresa [16]	Ireland	2010	Experimental	Rats	Bone marrow-derived mesenchymal tissue stem cells
Yang, Hao [17]	China	2019	Experimental	Male Balc/c mice	Testicular tissues from obstructive azoospermia patients at the age of 25–45 years
Edwards, George [18]	United States	2019	Experimental	MPTP-treated mice	Mouse embryonic tissue stem cells and mesenchymal stem cells
Tao, Yunlong [19]	United States	2021	Experimental	Rhesus monkeys	5 rhesus iPSC (RhiPSC) lines from monkeys
Pereira, Marcia [20]	United States	2022	Experimental	C57BL/6 J male mice (20–22 weeks old)	hNSCs derived from the ventricular zone in the telencephalon of late first-trimester human foetal cadavers
Kim, Ka Young [21]	South Korea	2021	Experimental	Four mouse groups	Human adipose-derived stem cells (hASCs) using magnetic nanoparticles
Park, Hyunjun [22]	South Korea	2020	Experimental	Three groups of mice	Human adipose-derived stem cells (hASC)
Chen, Yong-Ren [23]	Taiwan	2020	Experimental	Rats	Stem cells derived from human exfoliated deciduous teeth
Lara-Rodarte, Rolando [24]	Mexico	2021	Experimental	Female Wistar rats	R1 mouse embryonic stem cells (mESCs)
Tsai, May-Jywan [25]	Taiwan	2021	Experimental	Rats	Human MSCs from bone marrow of normal donor
Sun, Zhengqin [26]	China	2022	Experimental	Mice	Fresh umbilical cord samples were obtained from normal spontaneous full-term delivery mothers
Salama, Mohamed [27]	Egypt	2017	Experimental	30 B57BL/6 mice	MSCs from mononuclear cell fraction of pooled bone marrow from healthy C57BL/6 mice.
Qi, Li [28]	China	2017	Experimental	Sprague–Dawley rats	Neural stem cells were derived from neonatal SD rats.
Precious, Sophie [29]	United Kingdom	2020	Experimental	Adult male mice C57/Bl6	Cells derived from primary foetal mouse tissue
Nesti, Claudia [30]	Italy	2010	Experimental	Pregnant CD1 mice	Cells derived from human dental pulps
Terraf, Panieh [31]	Iran	2015	Experimental	Mouse	Undifferentiated R1 [Passage 9, (P9)] feeder-dependent mouse ES cells
Hoban, Deidre [32]	United States	2020	Experimental	Rats	Human embryonic stem cells (hESCs)
Takahashi, Haruka [33]	Japan	2017	Experimental	Rats	Nerve cells derived from human buccal fat pad stem cells
Yan, Yu-Chen [34]	China	2023	Experimental	Mouse	Bone marrow-derived neuronal stem cells
Forouzandeh, Meysam [35]	Iran	2021	Experimental	Rats	MSCs isolated from human conjunctiva (CJ-MSCs)
Shroff, Geeta [36]	India	2015	Experimental	Human	hESCs derived from 2-cell staged fertilised spare ovum
Venkataramana, N.K. [37]	India	2012	Experimental	Human	BM-MSCs isolated from healthy donors (ages of 18–30)
Schiess, Mya [38]	United States	2021	Experimental	Human	Allogeneic bone marrow-derived mesenchymal stem cells
Shigematsu, Kazuo [39]	Japan	2022	Experimental	Human	Adipose tissue-derived mesenchymal stem cells (ADSCs)
Madrazo, I [40]	Mexico	2019	Experimental	Human	Human neural progenitor cells (NPCs) derived from foetal brain tissue
Zakerinia, M [41]	Iran	2018	Experimental	Human	Haematopoietic stem cell derived from bone marrow

## Data Availability

All data are available in the manuscript and Supplementary Materials.

## Supplementary Materials

The following supporting information can be downloaded at: https://www.mdpi.com/article/10.3390/cells14110822/s1, Supplementary Materials: Complete Search Strategy and Formulas; Results of individual sources of evidence, with information of the subject used in each study and the outcome.

## Abbreviations

The following abbreviations are used in this manuscript:

CENTRAL	Cochrane Central Register of Controlled Trials
GH	Growth hormone
IGF-1	Insulin-like growth factor 1
MeSH	Medical Subject Headings
NHLBI	National Heart, Lung, and Blood Institute
PitNET	Pituitary neuroendocrine tumour
PRISMA	Preferred Reporting Items for Systematic Reviews and Meta-Analyses
PROSPERO	International Prospective Register of Systematic Reviews
VHL	Virtual Health Library
WHO	World Health Organization
