# Supplementary material for: Tissue Stem Cell-Based Therapies in Parkinson’s Disease: A Scoping Review of Therapeutic Mechanisms and Translational Outcomes
Source: Cells. 2025 Jun 1;14(11):822. doi: 10.3390/cells14110822 (PMC12154328; doi:10.3390/cells14110822)
Supplement: Supplementary file 1 [file cells-14-00822-s001.zip › cells-3635901-supplementary.pdf]

SUPPLEMENTARY MATERIALS

This supplementary document provides additional data to support the findings of the scoping review titled "Tissue Stem Cell-Based Therapies in Parkinson's Disease: A Scoping Review of Therapeutic Mechanisms and Translational Outcomes." It is divided into two main sections: (1) the complete search strategies and Boolean formulas used across the four databases (PubMed, Scopus, Cochrane, and the Virtual Health Library), and (2) a detailed table presenting individual study characteristics and outcomes, including type of model used (cell, animal, or human), tissue stem cell source, and therapeutic results. This structure is intended to facilitate transparency and reproducibility.

Complete Search Strategy and Formulas

Database	Strategy
**PubMed**	((Parkinson) OR (Parkinson's AND disease) OR (Idiopathic AND Parkinson's AND Disease) OR (Lewy AND Body AND Parkinson's AND Disease) OR (Parkinson's Disease, Idiopathic) OR (Parkinson's Disease, Lewy Body) OR (Parkinson Disease, Idiopathic) OR (Idiopathic AND Parkinson AND Disease) OR (Lewy AND Body AND Parkinson AND Disease) OR (Primary AND Parkinsonism) OR (Parkinsonism, Primary) OR (Paralysis AND agitans) OR ("Parkinson Disease" [MeSH])) AND ((Stem AND Cells) OR (Progenitor AND Cells) OR (Mother AND Cell) OR (Cell, Stem) OR (Cells, Stem) OR (Stem AND Cells) OR (Cell, Progenitor) OR (Cells, Progenitor) OR (Progenitor AND Cell) OR (Mother AND Cells) OR (Cell, Mother) OR (Cells, Mother) OR (Colony-Forming AND Unit) OR (Colony AND Forming AND Unit) OR (Colony-Forming AND Units) OR (Colony AND Forming AND Units) OR ("Stem Cells"[MeSH])) AND ((Disease AND Progression) OR (Disease AND progressions) OR (Progression, Disease) OR (Disease AND Exacerbation) OR ("Disease Progression" [MeSH]))
**Scopus**	TITLE-ABS-KEY (((Parkinson) OR (Parkinson's disease) OR (Parkinson disease) OR (Idiopathic Parkinson's Disease) OR (Lewy Body Parkinson's Disease) OR (Parkinson's Disease, Idiopathic) OR (Parkinson's Disease, Lewy Body) OR (Parkinson Disease, Idiopathic) OR (Idiopathic Parkinson Disease) OR (Lewy Body Parkinson Disease) OR (Primary Parkinsonism) OR (Parkinsonism, Primary) OR (Paralysis Agitans)) AND ((Stem Cells) OR (Cell, Stem) OR (Cells, Stem) OR (Progenitor cells) OR (Cell, Progenitor) OR (Cells, Progenitor) OR (Mother Cells) OR (Cell, Mother) OR (Cells, Mother) OR (Mother Cell) OR (Colony-Forming Unit) OR (Colony Forming Unit) OR (Colony-Forming Units) OR (Colony Forming Units) OR (Stem Cell)) AND ((Disease Progression) OR (Disease progressions) OR (Progression, Disease) OR (Disease AND Exacerbation)))
**Cochrane**	#1 Parkinson<br>#2 Parkinson Disease<br>#3 Parkinson's Disease<br>#4 Paralysis agitans<br>#5 MeSH descriptor:[Parkinson Disease]<br>#6 #1 OR #2 OR #3 OR #4 OR #5<br>#7 Stem Cells<br>#8 Progenitor Cells<br>#9 Mother Cells<br>#10 meSH descriptor: [Stem Cells]<br>#11 #7 OR #8 OR #9 OR #10<br>#12 Disease Progression<br>#13 Disease progressions<br>#14 Disease Exacerbation<br>#15 meSH descriptor: [Disease Progression]<br>#16 #12 OR #13 OR #14 OR #15<br>#17 #6 AND #10 AND #16
**VHL**	(((Parkinson Disease) OR (Idiopathic Parkinson Disease) OR (Idiopathic Parkinson's Disease) OR (Lewy Body Parkinson Disease) OR (Lewy Body Parkinson's Disease) OR (Paralysis Agitans) OR (Parkinson's Disease) OR (Parkinson's Disease Idiopathic) OR (Parkinson's Disease Lewy Body) OR (Parkinsonism Primary) OR (Primary Parkinsonism) OR (Enfermedad de Parkinson) OR (Enfermedad de Parkinson con Cuerpos de Lewy) OR (Enfermedad de Parkinson Idiopática) OR (Parálisis Agitante) OR (Parkinsonismo Primario)) AND ((Stem Cells) OR (Mother Cell) OR (Progenitor Cell) OR (Stem Cell) OR (Mother Cells) OR (Progenitor Cells) OR (Stem Cells) OR (Colony Forming Unit) OR (Colony Forming Units) OR (Colony-Forming Unit) OR (Colony-Forming Units) OR (Mother Cell) OR

	(Mother Cells) OR (Progenitor Cell) OR (Progenitor Cells) OR (Stem Cell) OR (Células Madre) OR (Células Básicas) OR (Células Primitivas) OR (Células Primordiales) OR (Células Progenitoras) OR (Células Troncales) OR (Unidades que Forman Colonias)) AND ((Disease Progression) OR (Disease progressions) OR (Progression, Disease) OR (Disease AND Exacerbation) OR (Curso de la enfermedad) OR (Progresion de la enfermedad) OR (progreso de la enfermedad) OR (avance de la enfermedad)))

Results of individual sources of evidence with information of the subject used in each study and the outcome.

First Author	Subject	Outcome
Acquarone	Mice	Mytomicin C treatment allowed pluripotent stem cells to restore motor function without forming tumors for as long as 15 months in mice
Calice Da Silva	Mice	Intrastriatal bone marrow mononuclear cells, but not intravenous, have a short-term therapeutic effect on the dopaminergic response of parkinsonian mice assessed by the apomorphine-induced rotation test.
Cerri	Rats	Delivery of Mesenchymal stem cells to the brain of 6-OHDA-lesioned animals can be obtained only after mannitol pretreatment, indicating that a permeabilizing agent is essential to allow passage across the blood-brain barrier.
Chen	Rats	Human exfoliated deciduous teeth exhibit promising efficacies in ameliorating neuroinflammation, α-synuclein clearance, the recovery of mitochondrial damage, and improving motor deficits in a rotenone-induced rat model of Parkinson's Disease. Intravenous administration of an adequate and single dose can improve the neurological outcome in PD rats significantly.
Edwards	Mice	Peripheral administration of stem cell-derived neural precursors obtained from mouse embryonic and mesenchymal stem cells may be a promising and safe therapy for the recovery of impaired motor function and amelioration of brain pathology in PD.
Forouzandeh	Rats	MSCs isolated from human Conjunctiva therapy can have protective effects against PD complications and nerve induction of cells due to their ability to express dopamine.
Hoban	Humanized α-synuclein rat model of PD	Transplanted human embryonic stem cells-derived DA neurons in this model proved they could survive, innervate, and integrate into host circuitry but that some grafted cells acquired the α-synuclein pathology at later time points.
Khademizadeh	Adipose derived stem cells	Human adipose tissue-derived stem cells can be a source of pluripotent MSCs able to differentiate to dopaminergic neurons simply and directly.
Kim	Mice	Motor function in PD model mice was found to recover via targeted distribution of human adipose stem cells using magnetic nanoparticles.
Kriks	Mice, rats, monkey	Long-term engraftment in 6-hydroxy-dopamine-lesioned mice and rats demonstrates robust survival of midbrain DA neurons derived human embryonic stem (ES) cells, complete restoration of amphetamine-induced rotation behavior and improvements in tests of forelimb use and akinesia.<br><br>Transplantation into Parkinsonian monkeys proved DA neuron survival and function.

Lara-Rodarte	Mice	Transgenic glial cell line-derived neurotrophic factor does not affect the pluripotency of mesenchymal stem cells and the sustained GDNF release increases the number of TH-positive neurons.<br><br>GDNF stem cells induced behavioral recovery and DA release in the brains of lesioned animals and increased the number of surviving dopamine neurons in the brains of a rodent model of PD.
Lian	Mice	PTX3, a hADSC-secreted protein, potentially protected the dopaminergic neurons against apoptosis and degeneration during PD progression and improved motor performance in PD mice.
Madrazo	Patients with Parkinson's disease with tremor, rigidity, or hypokinesia as major symptoms	At 4 years post-transplantation of brain grafting of human neural progenitor cells (NPCs) was reported that undifferentiated NPCs can be delivered by stereotaxis bilaterally to the putamina of patients with moderate to severe PD, without any complications attributable to the procedure. None of the patients showed unwanted motor disturbances (dyskinesias), tumor formation, or any detectable immune responses to the grafted cells.<br><br>In six out of seven cases, the procedure resulted in improved motor function, and a better response to L-DOPA and suggesting that at the very least the NPCs can stop or slow down the motor deterioration one would expect to see in this timespan.
May-Jywan Tsai	Rats	Transplantation of human mesenchymal stem cells alone or in combination with GDNF might provide trophic molecules to promote neuronal cell survival and foster a beneficial microenvironment that supports the repair of the nigrostriatal DA system.
Moloney	Rats	GDNF-transduced MSCs were capable of inducing a pronounced local trophic effect in the denervated striatum which was evident by sprouting from the remaining dopaminergic terminals towards the neurotrophic milieu created by the transplanted cells but this localised trophic effect was not sufficient to reinnervate the entire striatum.
Moreno	Human neuroepithelial cells	It successfully differentiated human neuroepithelial stem cells into dopaminergic neurons and after 30 days of differentiation, in situ morphological, immunohistological, electrophysiological characterization of dopaminergic neurons confirmed the biological fidelity of this new in vitro model and emphasized the biocompatibility of this microfluidic device.
Narbute	Rats	Extracellular vesicles (EVs) derived from human exfoliated deciduous teeth stem cells were administered intranasally in model rats of Parkinson’s disease into the medial forebrain bundle. It was demonstrated that EVs can effectively suppress 6-OHDA-induced gait impairments and normalize tyrosine hydroxylase expression in the striatum and in the substantia nigra of experimental rats.
Nesti	Mice	Dental pulp stem cells' protective effect on DA neurons was proved by immunocytochemistry, an increased number of spared tyrosine hydroxylase (TH)+ cells were observed in co-culture conditions compared to controls, and neurons showed longer processes in comparison with mesencephalic cells grown without DPSC.

Oh	Mesenchymal stem cells	MSCs exert neuroprotective properties through proteolysis of aggregated α-synuclein into soluble forms, and matrix metalloproteinase-2 may be the principal soluble factor released by MSCs responsible for proteolysis of α-synuclein aggregates.
Park	Mice	Repeated intravenous transplantation of human adipose stem cells may exert therapeutic effects on PD by restoring brain-derived neurotrophic factor and GDNF expressions, protecting dopaminergic neurons, and maintaining the nigrostriatal pathway.
Pereira	Mice	Neuron stem cells transplantation during the asymptomatic phase of PD may limit or halt the progression of this neurodegenerative disorder.
Precious	Mice	Dopaminergic transplants derived from two progenitor cell sources in an allograft system: mouse epiblast stem cells (EpiSC) and primary fetal mouse ventral mesencephalon tissue. Functional improvements were demonstrated posttransplantation in some behavioral tests, with no difference in graft volume or the number of TH immuno-positive cells in the grafts of the two transplant groups.
Qi	Rats	Lithium chloride (LiCl) treatment could enhance the proliferation in neuron stem cells and promote the dopaminergic neuronal differentiation of NSCs in vitro.
Salama	Rats	The present study validated that the intranasal delivery of MSCs may be a potential safe, easy and cheap alternative route for stem cell treatment in neurodegenerative disorders.
Schiess	Subjects with mild/moderate Parkinson's disease	A single intravenous infusion of allogeneic bone marrow-derived mesenchymal stem cells at doses of 1, 3, 6, or 10 × 106 allogeneic bone marrow-derived mesenchymal stem cells/kg is safe, well tolerated, and not immunogenic in mild/moderate PD patients.
Shigematsu	Patients with Parkinson's disease	Repeated administration of Autologous adipose tissue stem cells for Parkinson's disease was safe and feasible.
Shroff	A single patient with Parkinson's disease	This study presents the clinical outcome of a patient with Parkinson's disease treated with human embryonic stem cell therapy. The patient demonstrated an improvement in his overall condition and symptoms related to PD.
Takahashi	Rats	It was demonstrated the differentiation of buccal fat pad into neural cells, and that transplantation of these neural cells improved the symptoms of model rats. Our results suggest that neurons differentiated from hBFP-ASCs would be applicable to cell replacement therapy of PD.
Terraf	Mice	PCL/Matrigel nanofibrous scaffolds could efficiently support and promote the generation of functional dopaminergic-like cells from Nurr1, an essential transcription factor in dopaminergic neuron development, and GPX-1 a neuroprotective enzyme against oxidative stress cells.
Venkataramana	Patients with Parkinson's disease	Bilateral allogenic transplantation of adult bone marrow-derived mesenchymal stem cells is safe and has beneficial neuroprotective and neurorestorative effects. There are improvements in the UPDRS scores of the PD patients, reported subjective well-being and no increase in medications during the follow-up period.

Yan	Mice	The current study proved that the combination of fasudil and NSCs has a synergetic effect on protecting neuron loss, accompanied by NSCs survival, proliferation and migration, M2 microglia polarization, and anti-inflammation as well as production of neurotrophic factors.
Yang	Human spermatogonial stem cells	Human spermatogonial stem cells could acquire dopaminergic neuron morphological features and functional properties and rescue parkinsonian phenotypes.
Yunlong	Monkeys	It was proved that over a 2-year period without immunosuppression PD monkeys receiving autologous, but not allogeneic, transplantation exhibited recovery from motor and depressive signs. These behavioral improvements were accompanied by robust grafts with extensive dopamine neuron axon growth as well as strong DA activity in Positron Emission Tomography.
Zakerinia	Patients with various neurological diseases	After the intrathecally thansplatation with each patient bone marrow stem cells it waas noted clinical improvements in 9 of 12 patients with Parkinson’s disease, 20 of 28 patients with cerebral palsy, 6 of 7 patients with hypoxic brain damage, 2 of 4 patients with multiple sclerosis, and 4 of 5 patients with cerebellar atrophy. The improvements were noted after 2–4 weeks of cell therapy.
Zhengqin Sun	Mice	Umbilical cord mesenchymal stem cells modulated microbial composition in a 1-methyl-4-phenyl-1,2,3,6tetrahydropyridine (MPTP, a toxic agent that selectively destroys nigrostriatal neurons but does not affect dopaminergic neurons elsewhere) induced PD mouse model. UCMSCs ameliorate motor dysfunction and repair degeneration of dopamine neurons by inhibiting activated glial cells, decreasing the release of proinflammatory cytokines, maintaining the normal mucous barrier, and restraining the expression of NF-lB. The brain-gut axis may be a potential mechanism underlying the beneficial effect of UC-MSCs on PD mice.
